# Pathologic response prediction to neoadjuvant chemotherapy utilizing pretreatment near-infrared imaging parameters and tumor pathologic criteria

**DOI:** 10.1186/s13058-014-0456-0

**Published:** 2014-10-28

**Authors:** Quing Zhu, Liqun Wang, Susan Tannenbaum, Andrew Ricci, Patricia DeFusco, Poornima Hegde

**Affiliations:** 1 Biomedical Engineering and Electrical and Computer Engineering Departments, University of Connecticut, 371 Fairfield Road, U2157, Storrs, 06269 CT USA; 2 Department of Statistics, University of Manitoba, 186 Dysart Road, Winnipeg, R3T 2N2 MB Canada; 3 Clinical and Translational Breast Program, University of Connecticut Health Center, 263 Farmington Avenue, Farmington, 06030 CT USA; 4 Department of Pathology, Hartford Hospital, 80 Seymour Street, Hartford, 06106 CT USA; 5 Helen & Harry Gray Cancer Center, Hartford Hospital, 80 Seymour Street, Hartford, 06106 CT USA

## Abstract

**Introduction:**

The purpose of this study is to develop a prediction model utilizing tumor hemoglobin parameters measured by ultrasound-guided near-infrared optical tomography (US-NIR) in conjunction with standard pathologic tumor characteristics to predict pathologic response before neoadjuvant chemotherapy (NAC) is given.

**Methods:**

Thirty-four patients’ data were retrospectively analyzed using a multiple logistic regression model to predict response. These patients were split into 30 groups of training (24 tumors) and testing (12 tumors) for cross validation. Tumor vascularity was assessed using US-NIR measurements of total hemoglobin (tHb), oxygenated (oxyHb) and deoxygenated hemoglobin (deoxyHb) concentrations acquired before treatment. Tumor pathologic variables of tumor type, Nottingham score, mitotic index, the estrogen and progesterone receptors and human epidermal growth factor receptor 2 acquired before NAC in biopsy specimens were also used in the prediction model. The patients’ pathologic response was graded based on the Miller-Payne system. The overall performance of the prediction models was evaluated using receiver operating characteristic (ROC) curves. The quantitative measures were sensitivity, specificity, positive and negative predictive values (PPV and NPV) and the area under the ROC curve (AUC).

**Results:**

Utilizing tumor pathologic variables alone, average sensitivity of 56.8%, average specificity of 88.9%, average PPV of 84.8%, average NPV of 70.9% and average AUC of 84.0% were obtained from the testing data. Among the hemoglobin predictors with and without tumor pathological variables, the best predictor was tHb combined with tumor pathological variables, followed by oxyHb with pathological variables. When tHb was included with tumor pathological variables as an additional predictor, the corresponding measures improved to 79%, 94%, 90%, 86% and 92.4%, respectively. When oxyHb was included with tumor variables as an additional predictor, these measures improved to 77%, 85%, 83%, 83% and 90.6%, respectively. The addition of tHb or oxyHb significantly improved the prediction sensitivity, NPV and AUC compared with using tumor pathological variables alone.

**Conclusions:**

These initial findings indicate that combining widely used tumor pathologic variables with hemoglobin parameters determined by US-NIR may provide a powerful tool for predicting patient pathologic response to NAC before the start of treatment.

**Trial registration:**

ClincalTrials.gov ID: NCT00908609 (registered 22 May 2009)

**Electronic supplementary material:**

The online version of this article (doi:10.1186/s13058-014-0456-0) contains supplementary material, which is available to authorized users.

## Introduction

Preoperative or neoadjuvant chemotherapy (NAC) is increasingly used in the management of locally advanced breast cancers, as well as in patients with lower tumor stages, to increase the rate of breast-conserving therapy and to reduce the extent of surgery [1]-[3]. Complete eradication of invasive tumor cells in the primary tumor bed following neoadjuvant therapy is strongly correlated with improved disease-free survival and overall survival [4]. Furthermore, clinical trials in the NAC setting are increasingly being conducted to study new agents and novel therapeutic strategies in breast cancer using pathological complete response (pCR), a surrogate marker for survival, as the primary endpoint [5]. Several pathologic variables, such as invasive ductal carcinoma, high tumor grade and high proliferative activity, are associated with a better response to NAC [6]-[8]. Classifying breast cancers into molecular subtypes has significantly improved the understanding of preoperative chemotherapy outcomes and has helped guide the selection of treatment [9]-[11]. Recent studies have established that breast cancers that are basal-like or “triple-negative” (estrogen receptor–negative (ER–), progesterone receptor–negative (PR–) and human epidermal growth factor receptor 2–negative (HER2–)) respond best to cytotoxic therapies [11],[12] and that HER2-positive (HER2+) tumors respond best to trastuzumab-based regimens [13]. In particular, in HER2+ breast cancer, the NAC approach has yielded great successes. The dual HER2 blockade with trastuzumab and pertuzumab recently has shown the highest pCR rates ever reported [3]. The NAC approach has yielded much higher rates of pCR in patients with triple-negative breast cancers than for patients with other breast tumor types. However, more than half of triple-negative breast cancer patients do not achieve a pCR and have a very poor prognosis [14]. Current studies are focused on identifying molecular subtypes of triple-negative tumors and their clinical relevancy by determining pCR rates after NAC [15],[16]. Recent studies have also shown that luminal A subtype tumors (ER + and HER2− and low tumor grade or low-proliferative phenotype) exhibit lower sensitivity to standard cytotoxic-based regimens [17].

Nomograms, which integrate clinical and pathological variables including tumor receptors and number of chemotherapy courses using multiple logistic regression model, have been developed to predict complete pathological response on the basis of preoperative treatment [18]-[20]. However, reliable individualized prediction of a pathological complete response after preoperative chemotherapy based on conventional pathologic tumor characteristics determined before the start of treatment is difficult, and the response to chemotherapy varies among patients [21],[22].

In the past decade, optical tomography and optical spectroscopy using near-infrared (NIR) diffused light has demonstrated great potential in the assessment of the tumor vasculature response to NAC [23]-[30]. The NIR technique utilizes intrinsic hemoglobin contrast, which is directly related to tumor angiogenesis, a key process required for tumor growth and metastasis. In our recent paper published in *Radiology*[30], we demonstrated, for the first time to our knowledge, that the baseline pretreatment total hemoglobin (tHb), oxygenated hemoglobin (oxyHb) and deoxygenated hemoglobin (deoxyHb) levels were significantly higher in the tumors with near-complete or complete pathologic response than they were in the tumors with modest or no response to NAC. These measurements are directly related to tumor blood volume, perfusion, metabolism and tumor vasculature characteristics. Our new finding suggests that hypervascular tumors respond to NAC significantly better than hypovascular tumors do. In a recent study in which diffuse optical spectroscopy was used, Ueda *et al*. reported that the pretreatment tumor oxygen saturation = oxyHb/tHb × 100 correlated with pathological complete response for patients undergoing NAC [31]. To the best of our knowledge, our study and theirs are the only published ones in which prediction of NAC on the basis of pretreatment hemoglobin measurements has been described. Water has previously been reported to be sensitive to cell death, and its reduction may reflect a progressive loss of tumor cellularity and edema for at least 1 week [23],[32] or for 4 weeks [27] after initiation of NAC. Lipid and scatter measuring tumor tissue structure have not been reported as early predictors after the initiation of NAC, because more time may be needed before tumor size changes are detectable [23].

In this article, we introduce a novel prediction model using a multiple logistic regression model by incorporating widely used tumor pathologic variables of tumor type, grade and mitotic index, tumor receptors (triple-negative, HER2+ versus HER2−, ER− versus ER2+) and pretreatment functional parameters of tHb, oxyHb and deoxyHb. We assess the contributions of the hemoglobin functional parameters on improving the prediction sensitivity, specificity, positive predictive value (PPV), negative predictive value (NPV) and area under the receiving operating characteristic (ROC) curve (AUC), and we compare these measures with and without those obtained from conventional tumor pathologic characteristics.

## Methods

### Patients

Patients were recruited from Hartford Hospital and the University of Connecticut Health Center (UCHC) from December 2007 to May 2011. The study protocol was approved by the institutional review board of the Human Subjects Protection Office of UCHC and Hartford Hospital and is HIPAA-compliant (the Health Insurance Portability and Accountability Act). Written informed consent was obtained from all patients. Details of the patient study were reported previously [30]. Briefly, 32 patients who were treated with neoadjuvant chemotherapy were assessed pretreatment, at the end of each treatment cycle and prior to surgery using ultrasound-guided near-infrared optical tomography (US-NIR). Two more patients who completed the same study procedures and excluded in the previous report [30] were included in this study because the present study is focused on assessing pretreatment prediction. One patient had an inflammatory breast cancer with very low measurable vascular content throughout the treatment, and the other was an elderly patient treated differently from the rest. Patients’ tumor types, grades (Nottingham score), mitotic index scores and receptor status of ER, PR and HER2 obtained at core biopsy are summarized in Table 1 with the two patients discussed above listed at the bottom of the table.

**Table 1:** **Patient demographics** ^**a**^

Age, yr	Tumor type (IDC, 1) (IDC/ILC, 1) (ILC, 0 )	Nottingham score (out of 9)	Mitotic count/10 hpf	Triple-negative (+, 1) (−, 0)	HER2 (+, 1) (−, 0)	ER (+, 0) (−, 1)	Miller-Payne grade
32	1	9	20	0	0	0	2
51	1	8	8	0	0	0	2
42	1	8	8	1	0	1	5
64	1	9	34	1	0	1	5
48	1	4	0	1	0	1	3
34	1	6	2	0	0	0	1
39	0	7	1	0	0	0	2
48	1	5	6	0	0	0	1
39	1	9	14	1	0	1	1
42	0	3	0	0	0	0	1
35	1	9	39	0	0	0	4
40	1	4	2	0	0	0	2
47	1	9	15	0	0	0	5
53	0	6	0 or 1	0	0	0	2
32	1	9	16	0	1	1	4
64	1	6	4	0	1	1	4
40	1	9	30	0	1	0	3
38	1	7	9	0	1	0	4
64	1	8	44	0	0	0	2
48	0	6	1	0	0	0	2
69	1	7	16	0	0	0	3
82	1	6	0	0	0	0	1
47^b^	1	8	10	0	0	0	5
38	1	9	20	1	0	1	5
49	1	6	8	0	0	0	3
63^b^	1	8	10	0	0	0	3
37	1	7	8	0	0	0	4
55	1	7	10	0	1	0	5
44	1	6	5	0	0	0	3
53	1	9	58	1	0	1	5
54	1	9	26	0	0	1	5
42	0	6	5	0	0	0	2
77	1	9	16	0	1	0	5
35	1	6	4	0	0	0	3

^a^ER, Estrogen receptor; HER2, Human epidermal growth factor receptor 2; hpf, High-power fields; IDC, Invasive ductal carcinoma; IDC/ILC, Invasive mammary carcinoma with mixed ductal and lobular features; ILC, Invasive lobular carcinoma. ^b^Two distinct tumors in the same breast with the same characteristics.

The 34 total patients (mean age, 48 years; range, 32 to 82 years) were initially split into a training group of 23 patients with a total of 24 tumors enrolled into this study during the first 3 years of the recruitment period and a testing group of 11 patients with a total of 12 tumors. Of these latter 11 patients, 9 patients were enrolled in the last 7 months of the recruitment period and two more were enrolled as discussed in the paragraph above. Thus, two-thirds of the patients are being used for training and one-third for testing. Owing to the small patient sample, especially the limited numbers of triple-negative tumors (*n* = 6), HER2+ tumors (*n* = 6) and invasive lobular carcinomas (ILCs) (*n* = 5), we performed cross-validation by randomly splitting the six triple-negative tumors, six HER2+ tumors and five ILCs between training and testing data sets while keeping approximately two-thirds of the samples of each subcategory in the training data sets. So, 24 pairs of training and testing data sets were generated. Additionally, the rest of the patients were randomly split into training and testing data sets to generate six more pairs of training and testing data sets while keeping approximately a similar percentage (30% to 50%) of patients who achieved a complete or nearly complete response to NAC in each pair of training and testing data sets. Thus, a total of 30 pairs of training and testing data sets were generated to train, validate and compare the prediction models.

Among the 34 patients, 28 HER2- patients were treated with paclitaxel-based regimens (dose-dense doxorubicin/cyclophosphamide/paclitaxel, docetaxel/cyclophosphamide, doxorubicin/cyclophosphamide/docetaxel, and bevacizumab), and 6 HER2+ patients were treated with a trastuzumab-based regimen (docetaxel/carboplatin with trastuzumab). The final pathologic response was assessed using the Miller-Payne grading system [33], in which pathologic response is divided into five grades based on comparison of tumor cellularity between pre-neoadjuvant core biopsy and definitive surgical specimen. The Miller-Payne grading system is as follows:

- Grade 1: no change or some minor alteration in individual malignant cells, but no reduction in overall cellularity
- Grade 2: a minor loss of tumor cells, but overall high cellularity; up to 30% reduction of cellularity
- Grade 3: between an estimated 30% and 90% reduction in tumor cellularity
- Grade 4: a marked disappearance of more than 90% of tumor cells such that only small clusters or widely dispersed individual cells remain (almost pCR)
- Grade 5: no invasive malignant cells identifiable in sections from the site of the tumor (pCR)

For this study, the Miller-Payne grades 4 and 5 patients were grouped as responders and grades 1 to 3 were groups as nonresponders. There were a total of 21 grades 1 to 3 tumors and 15 grade 4 or 5 tumors with a response rate of 42%.

### Hemoglobin parameters

The imager consisted of a handheld probe with nine source fibers and ten detection light guides deployed around a commercial US probe. The US images were used to localize the tumor and were acquired simultaneously with the NIR data. For each patient, tumor absorption maps obtained at four optical wavelengths of 740, 780, 808 and 830 nm were reconstructed. From the absorption maps, the tHb, deoxyHb and oxyHb maps were calculated and the maximum tHb, deoxyHb and oxyHB concentrations were measured. Several quality NIR images at the tumor location were used to compute the average maximum tHb, deoxyHb and oxyHB values, which were used to characterize each tumor as reported elsewhere [30]. From phantom studies, we found that the reconstructed maximum value closely represented the true target value.

### Statistical analysis and prediction model

Spearman’s rank correlation coefficient or Spearman’s ρ, which is more appropriate for assessing the relationship for both continuous and discrete variables, was computed between each tumor’s Miller-Payne grade and the pretreatment maximum tHb, oxyHb and deoxyHb concentrations; tumor types; Nottingham scores; mitotic index; and tumor receptor status obtained at the core biopsy. Additionally, the Spearman’s ρ between pretreatment parameters was computed. Ductal carcinomas were coded as 1, mixed ductal and lobular carcinomas were coded as 1 and lobular carcinomas were coded as 0. Triple-negative, HER2 and ER tumor status was coded as 1 for triple-negative and 0 for otherwise, 1 for HER2+ and 0 for HER2−, and 0 for ER+ and 1 for ER−. Spearman’s correlation calculations were performed using Minitab 15 software (Minitab, State College, PA, USA), and the results are given in Table 2.

**Table 2:** **Statistical analysis of Miller-Payne grades compared with pretreatment variables** ^**a**^

	Maximum tHb	Maximum oxyHb	Maximum deoxyHb	Tumor type	NS	MC/10 hpf	Triple-negative	HER2	ER
Spearman’s ρ	0.520	0.455	0.395	0.408	0.545	0.538	0.261	0.305	0.375
*P*-value	0.001	0.005	0.017	0.013	0.001	0.001	0.124	0.070	0.024

^a^deoxyHb, Deoxygenated hemoglobin; ER, Estrogen receptor; HER2, Human epidermal growth factor receptor 2; hpf, High-power fields; MC, Mitotic count; NS, Nottingham score; oxyHb, Oxygenated hemoglobin; tHb, Total hemoglobin.

Logistic regression is a statistical modeling approach that can be used to describe the relationship of several predictor variables, *X*_1_, *X*_2_, … *X*_k_, to a dichotomous response variable *Y*, where *Y* is coded as 1 (responder) or 0 (nonresponder) for its two possible categories [34]. The model can be written in a form that describes the probability of occurrence of one of the two possible outcomes of *Y* as follows:

$$pr\left(Y\;=\;1\;|\;X1,\;X2,\;\ldots \;Xk\right)\;=\;\frac{1}{1\;+\;\exp\;(-\left(\beta 0\;+\;\Sigma_{n\;=\;1}^{k}\;\mathrm{\beta nXn}\right)}$$

The estimated outputs (probability) for each set of predictor variables range from 0 to 1. The model belongs to the class of generalized linear models based on the exponential distribution family. Given the data for *Y*, *X*_1_, *X*_2_, … *X*_k_, the unknown parameters βn, *n* = 0, 1, …, *k* can be estimated using the maximum likelihood method. In this article, we estimate and validate the 13 logistic models and their prediction power using combinations of 12 sets of predictor variables of tumor characteristics (tumor type, Nottingham score and mitotic counts), tumor pathological variables (tumor characteristics and receptor status of triple-negative, HER2 and ER), five pairs of hemoglobin predictor variables of tHb, oxyHb and deoxyHb only, tHb and oxyHb (tHboxyHb), and tHb and deoxyHb (tHbdeoxyHb), without tumor pathological variables and with these variables. The MATLAB (version 2008a; MathWorks, Natick, MA, USA) logistic regression function glmfit was used to compute the coefficients βn, where *n* = 0, 1, …, *k*, and glmval was used to predict the response from these coefficients for the training set. The same coefficients obtained from the training set were used to predict the response for the testing set.

We also assess the overall performance of the prediction models through the ROC curves and the AUC for all training and testing sets. For this purpose, the estimated outputs ranging from 0 to 1 were inputted to the R console (version R 2.15.2; The R Project for Statistical Computing, Vienna, Austria), and the R open source software package pROC [35] was used in the R console to compute the ROC curve and AUC for each model using combined predictor variables. The 95% confidence interval (CI) of each AUC was obtained using 10,000 stratified bootstrap replicates, and the average CIs obtained from the training and testing groups were computed. A threshold of 0.5 was used to separate responders (>0.5) from nonresponders (≤0.5) for each prediction model output and prediction sensitivity, specificity, PPV and NPV were calculated accordingly. In Minitab 15, a two-sample, two-sided *t*-test was used to calculate the statistical significance of differences in the sensitivity, specificity, PPV, NPV and AUC of different models. *P* <0.05 was considered statistically significant.

## Results and discussion

**Figure 1:**
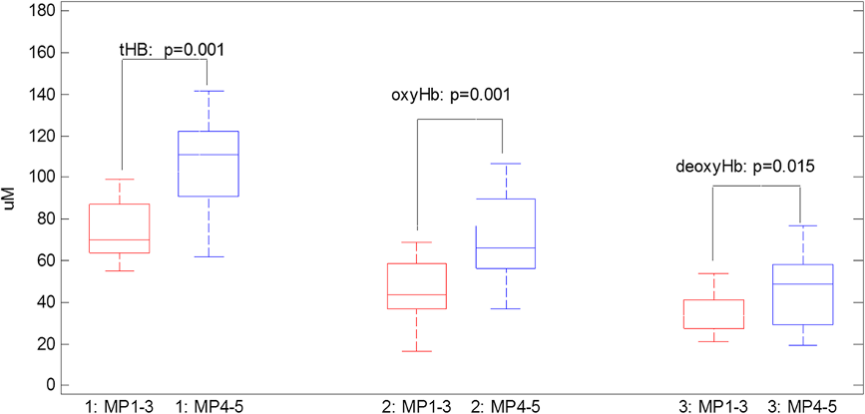
**Box-and-whisker plot of baseline total hemoglobin, oxygenated hemoglobin and deoxygenated hemoglobin of two responder groups.** deoxyHb, Deoxygenated hemoglobin; MP, Miller-Payne grade; oxyHb, Oxygenated hemoglobin; tHb, Total hemoglobin.

A box-and-whisker plot of pretreatment maximum tHb, oxyHb and deoxyHb values obtained from responder and nonresponder groups is shown in Figure 1. The corresponding mean values (SD) of pretreatment maximum tHb, oxyHb and deoxyHb values were 107.9 ± 33.2 μmol/L, 70.3 ± 23.1 μmol/L and 46.5 ± 17.6 μmol/L, respectively, for responders. The corresponding values were 72.8 ± 22.5 μmol/L, 45.5 ± 17.6 μmol/L and 34.1 ± 11.5 μmol/L for nonresponders (*P* = 0.001, *P* = 0.001 and *P* = 0.017, respectively) (Figure 1). The Spearman’s correlation coefficients of these parameters with Miller-Payne grades are summarized in Table 2. The maximum tHb, deoxyHb and oxyHb values correlate well with the final pathological response. Correlation coefficients of the other pathological predictor variables with Miller-Payne grades are also summarized in Table 2. As shown in the table, tumor type, Nottingham score, mitotic count and ER status correlate well with the final pathological response, and the triple-negative and HER2+ tumors also show reasonable correlation with the final pathological response. Patient age was not correlated with the Miller-Payne grade (Spearman’s ρ = 0.127, *P* = 0.473) and was not used as a predictor variable.

The Spearman’s correlation coefficients between pretreatment hemoglobin parameters and tumor pathological variables are given in Table 3. It is interesting to note that tHb and deoxyHb correlate well with tumor Nottingham score (*P* = 0.009 and *P* = 0.033, respectively) and that tHb and oxyHb show a moderate negative correlation with tumor ER expression (*P* = 0.064 and *P* = 0.049, respectively). Note that ER+ was coded as 0, and ER− was coded as 1. These results suggest that tumor hemoglobin levels estimated using the US-NIR imager measure the aggressiveness of breast cancers.

**Table 3:** **Statistical analysis of pretreatment hemoglobin parameters with tumor pathological variables** ^**a**^

	tHb	oxyHb	deoxyHb	Tumor type	NS	MC/10 hpf	Triple-negative	HER2	ER
tHb		0.841	0.637	0.166	0.430	0.206	0.108	0.144	0.312
		*P* < 0.001	*P* < 0.001	*P* = 0.333	*P* = 0.009	*P* = 0.229	*P* = 0.532	*P* = 0.404	*P* = 0.064
oxyHb			0.302	0.066	0.234	0.005	0.115	0.108	0.330
			*P* = 0.074	*P* = 0.703	*P* = 0.170	*P* = 0.979	*P* = 0.505	*P* = 0.532	*P* = 0.049
deoxyHb				0.050	0.356	0.292	0.093	0.086	0.275
				*P* = 0.771	*P* = 0.033	*P* = 0.084	*P* = 0.589	*P* = 0.618	*P* = 0.105

^a^deoxyHb, Deoxygenated hemoglobin; ER, Estrogen receptor; HER2, Human epidermal growth factor receptor 2; hpf, High-power field; MC, Mitotic count; NS, Nottingham score; oxyHb, Oxygenated hemoglobin; tHb, Total hemoglobin.

**Figure 2:**
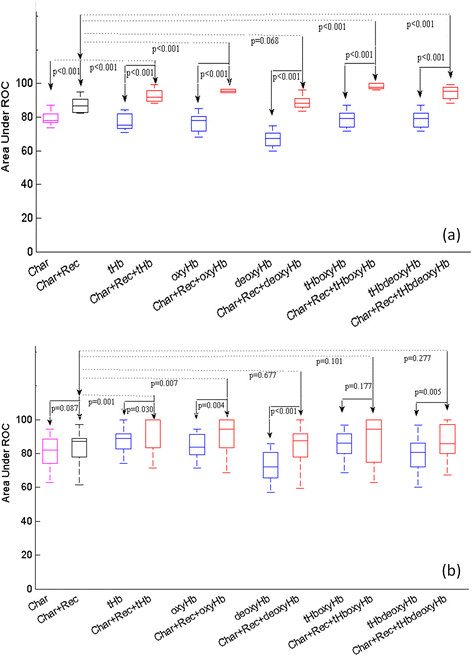
**Box-and-whisker plots of area under the receiver operating characteristic curves obtained from prediction models of 12 sets of predictor variables. (a)** Training data. **(b)** Testing data. Char, Predictor variables of tumor characteristics of type, Nottingham score, mitotic count; Char+Rec, Predictor variables of tumor characteristics and receptor status of triple-negative, human epidermal growth factor receptor 2 (HER2), estrogen receptor (ER); tHb, oxyHb, deoxyHb: Predictor variables of pretreatment maximum total hemoglobin (tHb), oxygenated hemoglobin (oxyHb) and deoxygenated hemoglobin (deoxyHb), respectively; tHboxyHb, tHbdeoxyHb: Combined predictor variables of tHb and oxyHb, tHb and deoxyHb, respectively; Char+Rec+corresponding hemoglobin variables: Combined predictor variables of tumor characteristics, receptor status and corresponding hemoglobin predictors. ROC, Receiver operating characteristic.

AUC statistics obtained from two sets of predictor variables of tumor characteristics (tumor type, Nottingham score and mitotic count), tumor pathological variables (tumor characteristics and receptor status of triple-negative, HER2, ER) are shown in the first two columns of Figure 2. The horizontal axis indicates the predictor variables. On average, the addition of the tumor receptor status improves the AUC from 76.9% (95% CI, 59.5; 95.46) to 87.1% (95% CI, 71.54; 98.63) in the training data (*P* <0.001) (Figure 2a) and from 80.0% (95% CI, 51.11; 99.29) to 84.0% (95% CI, 57.16; 99.03) in the testing data (*P* = 0.087) (Figure 2b). Additionally, five pairs of hemoglobin predictor variables of tHb, oxyHb, deoxyHb, tHboxyHb and tHbdeoxyHb without tumor pathological variables and with these variables are shown in groups in Figure 2. The average percentage AUCs of training and testing results, as well as average 95% CIs of all 12 prediction models using a different set of predictor variables, are summarized in Tables 4 and 5. For training data shown in (Figure 2a), the addition of the tumor pathological variables to each set of hemoglobin predictors significantly improves the AUC and tightens up the 95% CI as compared with data obtained without the pathological variables (*P* ≤ 0.001). The AUCs obtained from the hemoglobin predictors with tumor pathological variables are significantly higher than those of the pathological variables alone (*P* <0.001), except deoxyHb and deoxyHb with the tumor pathological variables pair (*P* = 0.068), which approaches statistical significance. For the testing data shown in Figure 2b, the combined predictor set of tHb with tumor pathological variables and oxyHb with these variables outperform tHb and oxyHb alone (*P* = 0.030 and *P* = 0.004, respectively) and pathological variables alone (*P* = 0.001 and *P* = 0.007, respectively). On average, the AUCs of tHb and oxyHb with tumor pathological variables are 92.4% (95% CI, 79.42; 99.80) and 90.6% (95% CI, 74.36; 99.35), respectively, as compared with 87.3% (95% CI, 62.13; 99.82) and 84.0% (95% CI, 56.01; 100), respectively, obtained without the pathological variables. Note that the 95% CI is much tighter with the addition of pathological variables. However, the other three sets of hemoglobin predictors combined with tumor pathological variables did not achieve statistical significance as compared with the tumor pathological variables alone. One reason is that tHb is correlated with oxyHb and deoxyHb (see Table 3). However, oxyHb and deoxyHb are independent variables. Because the signal-to-noise ratio of estimated tHb and oxyHb is much higher than that of deoxyHb, the tHb and oxyHb pair and tHb and deoxyHb pair are more robust predictors than the oxyHb and deoxyHb pair and are used with and without pathological variables as predictors for analysis. Both sets of the tHb and oxyHb pair and the tHb and deoxyHb pair contain information about tumor oxygenated and deoxygenated blood distributions. The combined predictor deoxyHb with tumor pathological variables performs better than deoxyHb alone (*P* <0.001). The validation data suggest that the combined predictor variables of tHb or oxyHb with tumor pathological variables are strong predictors of a patient’s final pathological response and are more informative than the other three sets of combined predictors.

**Table 4:** **Training data set results** ^**a**^

Tumor pathologic variables	Receptors (core biopsy)	Pretreatment hemoglobin parameters	Sensitivity (%)/specificity (%)	PPV (%)/NPV (%)	AUC (%) (95% CI)
IDC/ILC, NS, MC			76.2/74.3	65.7/83.1	76.9 (59.60; 95.46)
IDC/ILC, NS, MC	TN, HER2, ER		71.1/80.7	70.5/81.6	87.1 (71.54; 98.63)
		tHb	58.1/84.5	69.9 /75.9	76.7 (53.01; 96.64)
		oxyHb	52.9/86.1	71.5/73.8	76.8 (54.60; 94.34)
		deoxyHb	59.3/81.8	68.1/75.9	67.4 (40.97; 90.09)
		tHb, oxyHb	60.3/87.9	77.1/77.5	78.6 (55.46; 97.08)
		tHb, deoxyHb	64.1/85.2	74.3/78.5	74.6 (55.58; 97.31)
IDC/ILC, NS, MC	TN, HER2, ER	tHb	80.5/87.6	80.8/87.5	92.9 (82.76; 99.70)
IDC/ILC, NS, MC	TN, HER2, ER	oxyHb	79.9 /91.4	85.2/87.9	96.1 (88.91; 100.0)
IDC/ILC, NS, MC	TN, HER2, ER	deoxyHb	73.0/82.0	72.9/82.9	88.9 (74.94; 98.94)
IDC/ILC, NS, MC	TN, HER2, ER	tHb, oxyHb	92.5/95.2	92.5/95.2	98.2 (93.68; 100.0)
IDC/ILC, NS, MC	TN, HER2, ER	tHb, deoxyHb	83.9/87.2	80.7/89.7	95.1 (86.94; 99.75)

^a^CI, confidence interval; deoxyHb, Deoxygenated hemoglobin; ER, Estrogen receptor; HER2, Human epidermal growth factor receptor 2; IDC, Invasive ductal carcinoma; IDC/ILC, Invasive mammary carcinoma with mixed ductal and lobular features; ILC, Invasive lobular carcinoma; MC, Mitotic count; NS, Nottingham score; oxyHb, Oxygenated hemoglobin; NPV, Negative predictive value; PPV, Positive predictive value; tHb, Total hemoglobin; TN, Triple-negative.

**Table 5:** **Testing data set results** ^**a**^

Tumor pathologic variables	Receptors (core biopsy)	Pretreatment hemoglobin parameters	Sensitivity (%)/specificity (%)	PPV (%)/NPV (%)	AUC (%) (95% CI)
IDC/ILC, NS, MC			51.8/68.1	57.9/63.3	80.0 (51.11; 99.29)
IDC/ILC, NS, MC	TN, HER2, ER		56.8/88.9	84.8/70.9	84.0 (57.16; 99.03)
		tHb	63.2/82.1	75.0/72.8	87.3 (62.13; 99.82)
		oxyHb	60.8/81.9	73.6/71.4	84.0 (56.01; 100.0)
		deoxyHb	52.2/89.6	80.4/69.1	72.2 (36.55; 97.67)
		tHb, oxyHb	64.4/82.1	75.2/73.4	85.0 (57.61; 99.82)
		tHb, deoxyHb	60.7/80.6	73.3/70.9	79.5 (56.32; 97.82)
IDC/ILC, NS, MC	TN, HER2, ER	tHb	78.7/93.6	89.5/85.9	92.4 (79.42; 99.80)
IDC/ILC, NS, MC	TN, HER2, ER	oxyHb	76.9/85.2	82.6/83.3	90.6 (74.36; 99.35)
IDC/ILC, NS, MC	TN, HER2, ER	deoxyHb	60.1/85.0	78.4/71.8	85.0 (58.78; 98.95)
IDC/ILC, NS, MC	TN, HER2, ER	tHb, oxyHb	73.2/84.9	81.8/81.7	88.6 (72.21; 97.51)
IDC/ILC, NS, MC	TN, HER2, ER	tHb, deoxyHb	71.5/84.3	79.5/79.1	85.0 (63.09; 99.53)

^a^CI, confidence interval; deoxyHb, Deoxygenated hemoglobin; ER, Estrogen receptor; HER2, Human epidermal growth factor receptor 2; IDC, Invasive ductal carcinoma; IDC/ILC, Invasive mammary carcinoma with mixed ductal and lobular features; ILC, Invasive lobular carcinoma; MC, Mitotic count; NS, Nottingham score; oxyHb, Oxygenated hemoglobin; NPV, Negative predictive value; PPV, Positive predictive value; tHb, Total hemoglobin; TN, Triple-negative.

**Figure 3:**
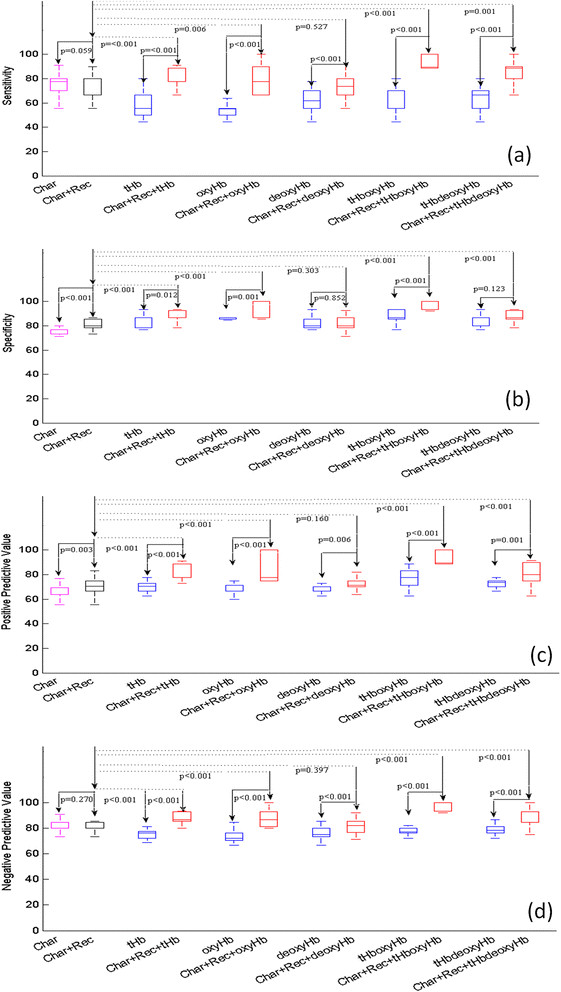
**Training data.** Box-and-whisker plot of sensitivity **(a)**, specificity **(b)**, positive predictive value **(c)**, and negative predictive value **(d)** obtained from 12 prediction models with predictor variables given along the *x*-axis. The predictor variables are the same as those given in Figure 2.

**Figure 4:**
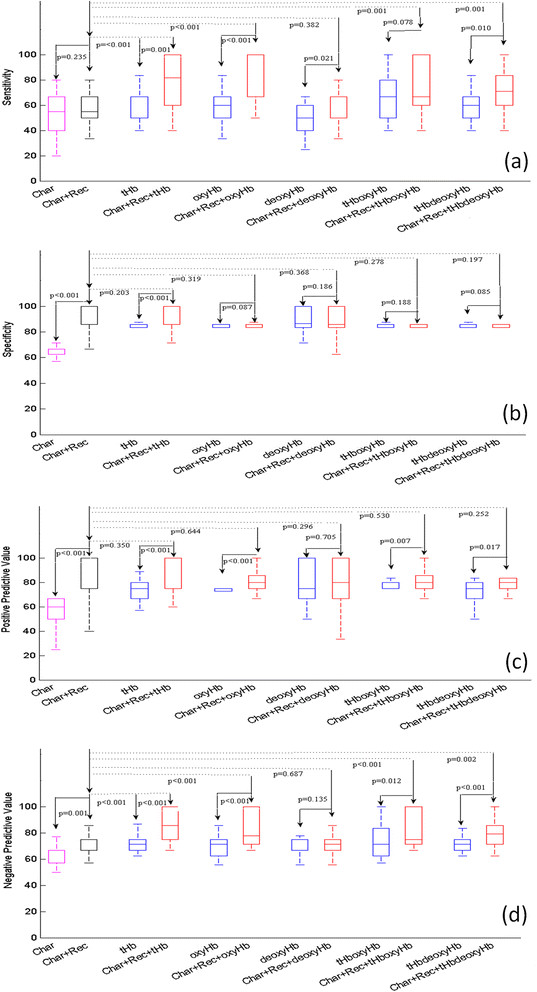
**Testing data.** Box-and-whisker plot of sensitivity **(a)**, specificity **(b)**, positive predictive value **(c)**, and negative predictive value **(d)** obtained from 12 prediction models with predictor variables given along the *x*-axis. The predictor variables are the same as those given in Figure 2.

Statistics of prediction sensitivity, specificity, PPV and NPV obtained from two sets of predictor variables of tumor characteristics, tumor pathological variables and five pairs of hemoglobin predictor variables without and with tumor pathological variables are shown in Figure 3 (training) and Figure 4 (testing), respectively. For the training data shown in Figure 3 and Table 4, three pairs of hemoglobin predictors of tHb, oxyHb, tHboxyHb combined with tumor pathological variables significantly improve prediction sensitivity, specificity, PPV and NPV as compared with corresponding hemoglobin predictors alone (*P* ≤ 0.012) and tumor pathological variables alone (*P* ≤ 0.006). deoxyHb combined with pathological variables does not improve the performance of these measures as compared with tumor pathological variables alone; tHbdeoxyHb combined with pathological variables does not improve specificity, but it does improve the other three measures. For the testing data shown in Figure 4 and Table 5, all hemoglobin predictors, except deoxyHb, combined with pathological variables significantly improve the prediction sensitivity and NPV as compared with tumor pathological variables alone (*P* ≤ 0.05). On average, the sensitivity and NPV of tHb, oxyHb, tHboxyHb, tHbdeoxyHb are 78.7% (*P* <0.001), 76.9% (*P* <0.001), 73.2% (*P* = 0.001), 71.5% (*P* = 0.001) and 93.6% (*P* <0.001), and 85.2% (*P* <0.001), 84.9% (*P* <0.001) and 84.3% (*P* = 0.002), respectively, as compared with 56.8% and 70.9% obtained from pathological variables alone. However, no statistically significant improvement is achieved in specificity and PPV as compared with prediction using tumor pathological variables alone. In general, all hemoglobin predictors combined with pathological variables perform better than hemoglobin predictors alone, except deoxyHb. tHb combined with the pathological variables is the best predictor, with an average 79% sensitivity, 94% specificity, 90% PPV and 86% NPV. The second best predictor is oxyHb combined with pathological variables, with corresponding measures of 77%, 85%, 83% and 83%, respectively.

**Figure 5:**
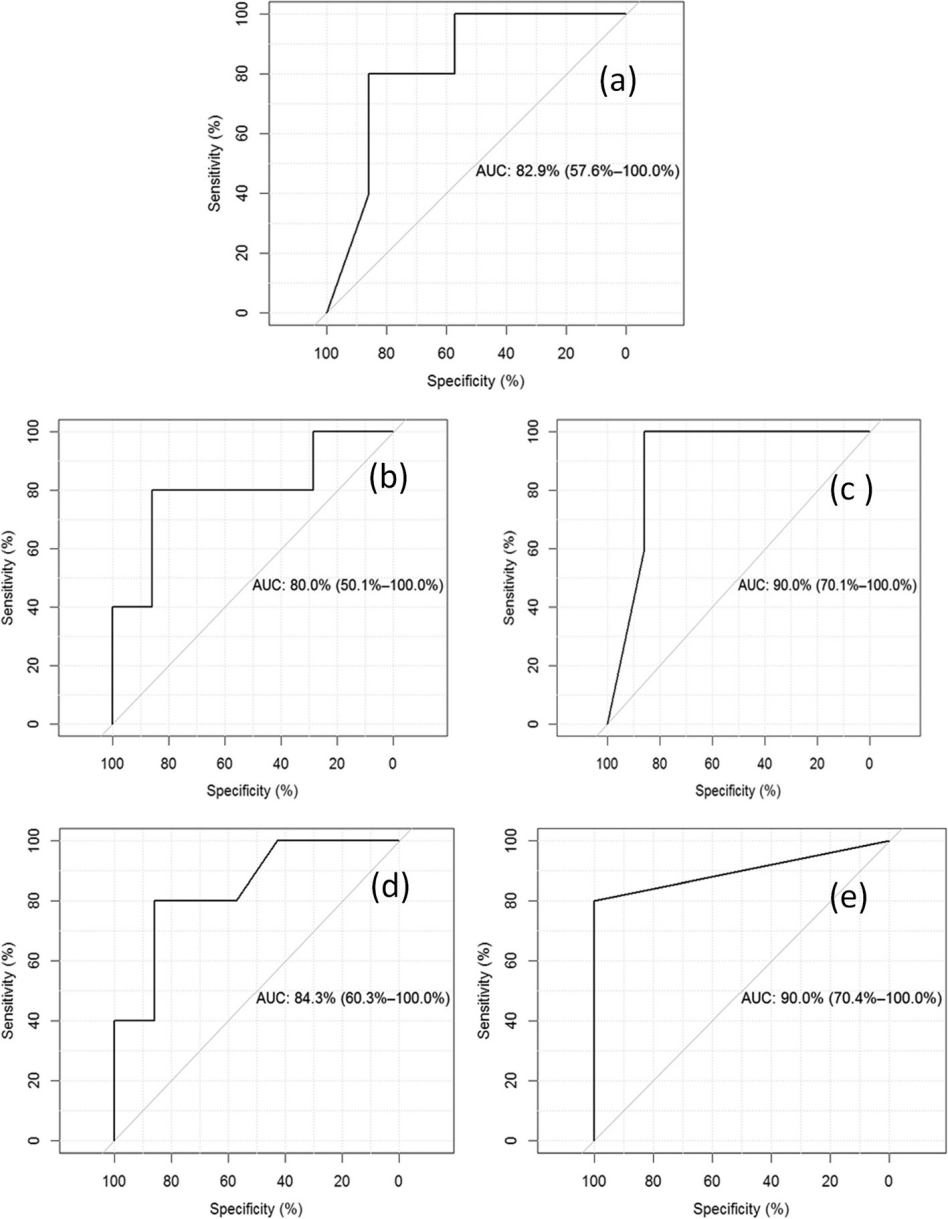
**Typical example of receiver operating characteristic curves obtained with pathological variables. (a)** Receiver operating characteristic curve (ROC) obtained from tumor pathological variables only. **(b)** ROC obtained from tumor tHb only. **(c)** ROC obtained from tHb with pathological variables. **(d)** ROC obtained from oxyHb only. **(e)** ROC obtained from oxyHb. The 95% confidence interval is also given in each figure.

Taken together, our findings based on AUC, sensitivity, specificity, PPV and NPV support the hypothesis that tHb and oxyHb combined with tumor pathological variables are strong pretreatment predictors of patient final pathological response. Figure 5 shows a typical example of ROC curves obtained from tumor pathological variables only (Figure 5a), tHb without pathological variables (Figure 5b) and tHb with pathological variables (Figure 5c), oxyHb without pathological variables (Figure 5d) and oxyHb with pathological variables. The AUC values computed by pROC are 82.9%, 80.0%, 90%, 84.3% and 90%, respectively. The 95% CI value is also given in each figure.

Different breast cancers have different degrees of chemotherapy sensitivity. Conventionally used tumor histopathological variables have been used to predict a patient’s pathological response. Immunohistochemistry results of ER, PR and HER2 status have been routinely evaluated in assisting and guiding the treatment selection. High tumor grade, ER−, triple-negative and HER2+ cancers have significantly higher rates of response to chemotherapy than other breast cancers. However, these models have always fallen short. Our data derived from 34 patients demonstrate that 67% of triple-negative tumors were Miller-Payne grade 5 and 83.3% of the HER2+ tumors were grade 4 or 5 (Table 6). On the basis of NIR pretreatment tHb level, we could predict responders with 80% accuracy for grades 4 and 5 tumors. It is known that ER+ tumors do not respond well to standard paclitaxel-based regimens. Our data show that 80.9% of the ER+ tumors were Miller-Payne grades 1 to 3. On the basis of NIR pretreatment tHb level, we could predict nonresponders with 69% accuracy for grades 1 and 2 tumors and 87.5% accuracy for grade 3 tumors (Table 6). This result is comparable to that based on receptor markers.

**Table 6:** **Distributions of tumor receptor status and tumor responses based on Miller-Payne grades** ^**a**^

	Miller-Payne grades 1 and 2	Miller-Payne grade 3	Miller-Payne grade 4	Miller-Payne grade 5
Triple-negative (*n* = 6)	*n* = 1 (17%, 1/6)	*n* = 1 (17%, 1/6)		*n* = 4 (67%, 4/6)
ER− HER2+ (*n* = 6)		*n* = 1 (16.7%, 1/6)	*n* = 3 (50%, 3/6)	*n* = 2 (33.3%, 2/6)
ER+ HER2− (*n* = 21)	*n* = 12(57.1%, 12/21)	*n* = 5(23.8%, 5/21)	*n* = 2 (9.5%, 2/21)	*n* = 2 (9.5%, 2/21)
ER− PR + HER2− (*n* = 1)				*n* = 1 (100%)
Pretreatment tHb > Th	*n* = 4 (31%, 4/13)	*n* = 1 (12.5%, 1/8)	*n* = 3 (60%, 3/5)	*n* = 9 (90%, 9/10)
Pretreatment tHb < Th^b^	*n* = 9 (69%, 9/13)	*n* = 7 (87.5%, 7/8)	*n* = 2 (40%, 2/5)	*n* = 1 (10%, 1/10)

^a^ER, Estrogen receptor; HER2, Human epidermal growth factor receptor 2; tHb, Total hemoglobin. ^b^Prediction based on pretreatment tHb. Th is the threshold used to separate responders (> Th = 90 μmol/L) from nonresponders (< Th). This threshold Th was selected in a previously reported study [30].

Our current study shows that when the pretreatment measurements of hemoglobin content are used together with histopathologic parameters as predictors in a multivariable prediction model, a substantially improved estimation of patient treatment outcome, especially prediction sensitivity and NPV, is obtained.

Ueda *et al*., using a diffuse optical spectroscopy technique, found that the pretreatment tumor oxygen saturation correlates with pathological complete response for patients undergoing NAC [31]. Our hemoglobin parameters were estimated from maximum values of US-guided tomographic images with spatial and depth distributions. In tomography, oxygen saturation = oxyHb/tHb × 100% can be obtained pixel by pixel using tHb as a denominator, and it is not robust for pixels with small tHb values. Therefore, we did not compute oxygen saturation directly; however, we show in Figure 1 that pretreatment tHb, oxyHb, deoxyHb predict responders from nonresponders with statistical significance.

ER− cancers are typically high-grade and more aggressive. Our study shows that tHb and oxyHb inversely correlate with ER expression. A related study was reported by Koukourakis *et al*., who found an inverse association of microvascular density with ER expression [36]. In another study [37], Fuckar *et al*. reported a negative correlation between vascular endothelial growth factor expression and ER status. These ER− negative tumors were characterized by higher proliferative activity. The precise mechanisms for oncogenic and angiogenic activities in ER− breast cancer are not fully understood [38].

This study has limitations. First, the patient sample is small, in particular the ILC, HER2+ and triple-negative tumor cases. A large sample size would make the logistic regression results more reliable because of the increased number of observations in each case and thus the increased accuracy in estimation of regression parameters and improved prediction. However, the predictive values of ILC, HER2+ and triple-negative tumors for NAC are well-documented in the literature, and the new knowledge reported in this study is the improved prediction of NAC by addition of hemoglobin parameters. Overfitting could occur when three hemoglobin predictors, as well as pretreatment optical scatter data, in addition to tumor pathological variables were used as predictor variables to fit a limited set of training data points. As a result, the model memorizes the training data and is less robust to generalize to an independent testing set. Currently, a larger-scale patient study is being designed to validate the initial results reported in this article. More data could allow robust estimation and validation of additional predictor variables, such as pretreatment optical scatter data and new biomarkers. Once validated with a larger patient pool, it may serve as a benchmark for preoperative chemotherapy prediction and also for integrating newly discovered molecular markers and guiding tailored therapy. Second, patients in the study were treated with standard chemotherapy regimens, including anthracyclines, taxanes and trastuzumab. The applicability of the prediction model to novel targeted agents remains to be tested in future clinical trials.

## Conclusions

As demonstrated by ROC analysis on testing data, tumor pathologic predictor variables achieved an average prediction sensitivity of 56.8%, specificity of 88.9%, PPV of 84.8%, NPV of 70.9% and AUC of 84.0%. tHb combined with the tumor pathological variables is the best predictor, with corresponding measures of 79%, 94%, 90%, 86% and 92.4%. oxyHb combined with pathological variables is the second best predictor, with corresponding measures of 77%, 85%, 83%, 83% and 90.6%. The addition of tHb or oxyHb significantly improves the prediction sensitivity, NPV and AUC as compared with using tumor pathological variables alone. Our initial data indicate that combining widely used breast tumor pathologic variables with novel tumor functional parameters of hemoglobin (assessed by using a US-NIR technique) as predictor variables may provide a powerful tool for predicting patient pathological response before the initiation of neoadjuvant chemotherapy.

## Authors’ original submitted files for images

Below are the links to the authors’ original submitted files for images. Authors’ original file for figure 1 Authors’ original file for figure 2 Authors’ original file for figure 3 Authors’ original file for figure 4 Authors’ original file for figure 5

## Abbreviations

AUC: Area under receiving operating characteristic curve
deoxyHb: Deoxygenated hemoglobin
ER: Estrogen receptor
HER2: Human epidermal growth factor receptor 2
NAC: Neoadjuvant chemotherapy
NPV: Negative predictive value
oxyHb: Oxygenated hemoglobin
PPV: Positive predictive value
PR: Progesterone receptor
ROC: Receiving operating characteristic curve
tHb: Total hemoglobin
TN: Triple-negative
