# Supplementary figures and images for: Pathologic response prediction to neoadjuvant chemotherapy utilizing pretreatment near-infrared imaging parameters and tumor pathologic criteria
Source: Breast Cancer Res. 2014 Oct 28;16:456. doi: 10.1186/s13058-014-0456-0 (PMC4303135; doi:10.1186/s13058-014-0456-0)

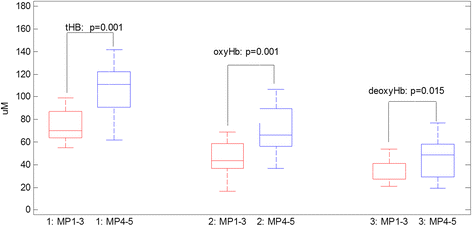

Supplement: Supplementary file 1 — Authors’ original file for figure 1 [file 13058_2014_456_MOESM1_ESM.gif]

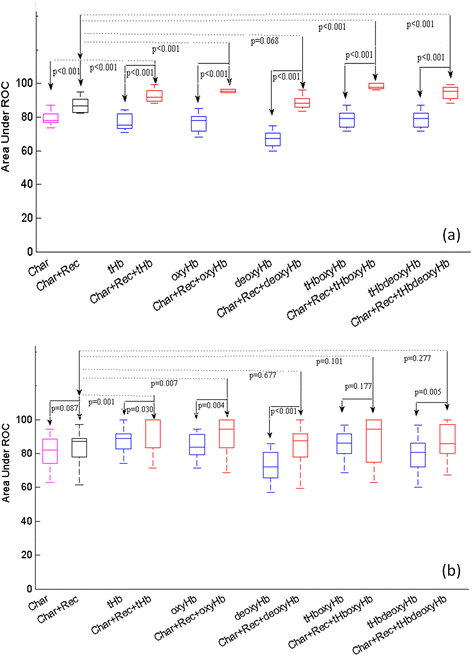

Supplement: Supplementary file 2 — Authors’ original file for figure 2 [file 13058_2014_456_MOESM2_ESM.gif]

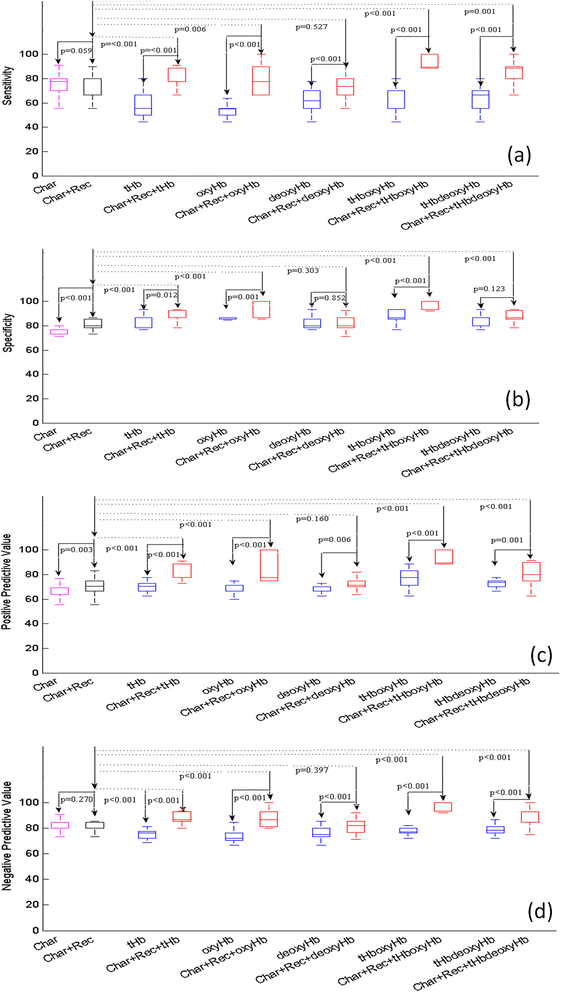

Supplement: Supplementary file 3 — Authors’ original file for figure 3 [file 13058_2014_456_MOESM3_ESM.gif]

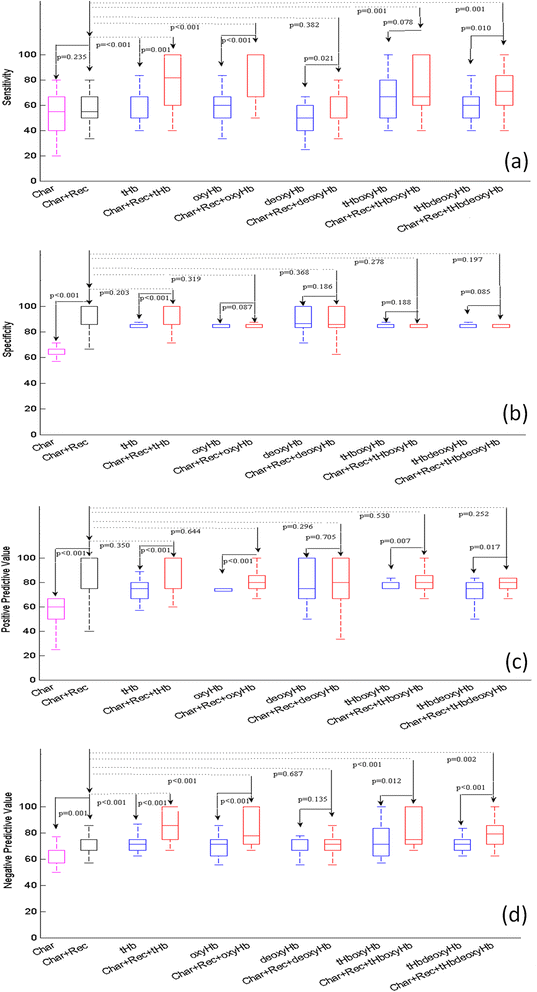

Supplement: Supplementary file 4 — Authors’ original file for figure 4 [file 13058_2014_456_MOESM4_ESM.gif]

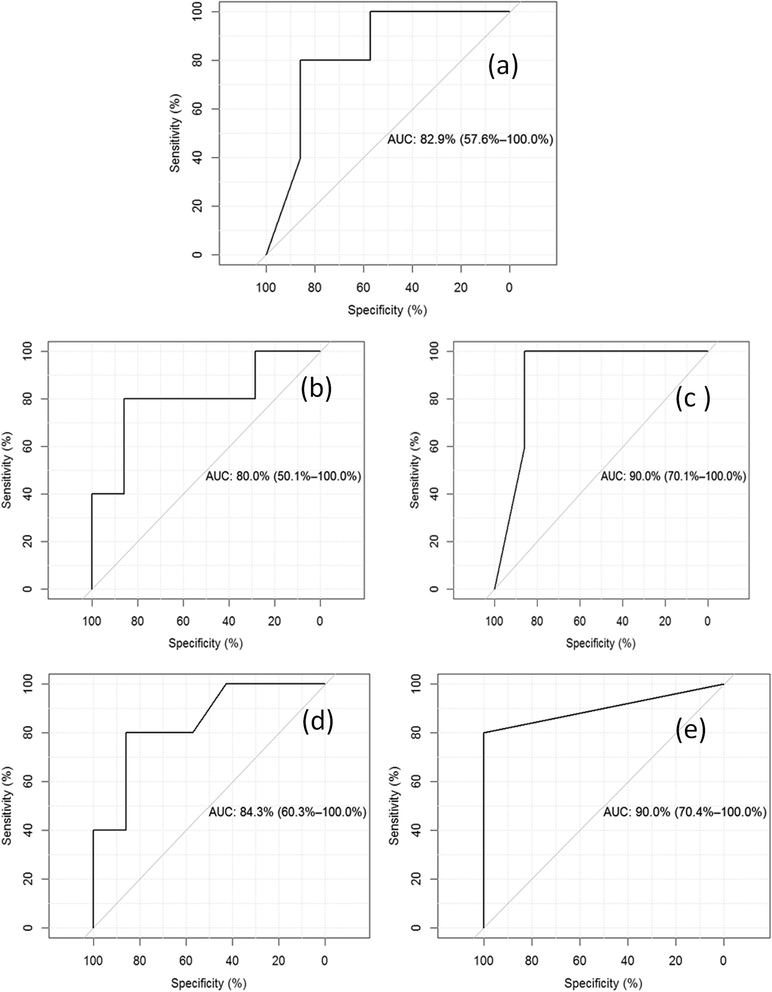

Supplement: Supplementary file 5 — Authors’ original file for figure 5 [file 13058_2014_456_MOESM5_ESM.gif]
